# Reports on the Hospitals of the United Kingdom

**Published:** 1910-04-16

**Authors:** Henry Burdett


### REPORTS

ON THE

### Hospitals of the United Kingdom.

By SIR HENRY BURDETT, K.C.B., K.C.V.O.

### XXXIX.—WORCESTER GENERAL INFIRMARY.

Our previous inspection of this infirmary was made immediately after the new sanitary blocks were added some 16 years ago. At the time these improvements were carried out so bad had been the previous state of affairs that the new blocks were hailed with satisfaction by all friends of the infirmary. To-day the blocks appear, as they undoubtedly are, small and lacking several essentials of modern sanitation. There is not room enough to do the work properly, or to provide a place for many requisites rendered essential to the efficient working of these large wards. The slop sinks have hot and cold water supplied to them, but experience teaches that a hot-water supply should never be brought into a slop sink if the health of the staff is taken into consideration. In the surgical wards new dressing trolleys should be provided, and the whole of the wards urgently need new lockers throughout.

The arrangements for the reception of casualties, and for the operations performed in this department, should be reorganised and perfected; a portable operation table of sufficient size should be provided.

### Mortuary and Ward.

The mortuary at present is not a credit to the hospital. The post-mortem room needs ventilation, and two inlets should be provided in it at the floor level. It also needs the attention of a painter. The mortuary proper does not exhibit due respect for the dead, and the sooner these matters are put in hand and corrected the better.

The large wards, which have not thorough cross-ventilation, should be reconstructed at once. Every window should be heightened by at least 3 feet, for in this way a wonderful change for the better in the hygienic condition of these wards would be secured.

We found the general good order and cleanliness of the hospital such that we were satisfied that the hospital is well served by its staff, who have to carry on much of their work at present under discouraging conditions. Having regard to the age of the hospital and the conditions to which we have referred, it reflects credit upon the resident staff that the wards are so well kept.

To our surprise, we found that the assistant matron has no sitting-room of her own, as this officer has in every well-organised hospital. Again, the sisters at this hospital have not their own table, and the accommodation for the nurses is inadequate. All meals should be served in the nurses' dining-room, and nowhere else. Cubicles, each having a window, are not satisfactory.

The hospital has certain nurses who have to sleep out, an arrangement which is never satisfactory, and which ought to be abolished by enlarging the nurses' home.

The laundry buildings were not planned by an architect who understood laundry management. All the linen enters by the same door, a very improper arrangement for a hospital. Indeed, the laundry as it stands, being without any machinery worked by electricity or steam, is altogether out of date and inadequate. The whole place needs reconstruction.

It is not, of course, possible to make these old buildings perfect, but if the suggestions we have ventured to make are carried out with promptness under the direction of someone who understands hospital construction thoroughly, the hospital will be greatly improved and benefited in every way at a relatively small cost.

### Accounts and Finance.

We note that the accounts of this hospital are not kept, as they ought to be, upon a uniform system. This is a matter which should receive the immediate attention of the House and Finance Committees, for by adopting the uniform system now used by all the best managed hospitals it will be possible to compare its working with that of similar institutions. So far as we can understand the accounts the expenditure would appear to be about £8,000 per annum. The invested funds exceed £60,000. Five years' expenditure amounts to £40,000, so that the committee have at present a margin of £20,000 out of which they can properly provide, if they so decide, the cost of a reconstruction and other matters we recommend.

The whole financial scheme of this hospital calls for prompt reform. The scale of privileges for governors is ridiculously low. One in-patient ticket for every guinea subscription; that is to say, that every man who gives £1 1s. to the hospital secures the right of taking £5 in value from its funds. This is neither philanthropy nor business, and the position is made infinitely worse from the fact that the deficiency in income in the last 25 years has amounted to £21,463, by which sum the invested funds have been diminished. Other county hospitals where the income was too small have reconsidered their scale of privileges for subscribers, with a great financial advantage. The Worcester General Infirmary authorities should set their finances in order at once, and we shall be very glad to help them if they decide to undertake promptly this important matter.

The **David Lewis Northern Hospital, Liverpool,** will be reported on next week.

